# Association between immune-mediated adverse events and efficacy in metastatic non-small-cell lung cancer patients treated with durvalumab and tremelimumab

**DOI:** 10.3389/fimmu.2022.1026964

**Published:** 2022-11-03

**Authors:** Agnish Dey, Matthew Austin, Harriet M. Kluger, Nataliya Trunova, Helen Mann, Norah Shire, Claire Morgan, Diansong Zhou, Ganesh M. Mugundu

**Affiliations:** ^1^ Clinical Pharmacology and Quantitative Pharmacology, Biopharmaceuticals R&D, AstraZeneca, Boston, MA, United States; ^2^ School of Medicine, Yale University, New Haven, CT, United States; ^3^ Immuno-Oncology Franchise, Oncology R&D, AstraZeneca, Gaithersburg, MD, United States; ^4^ Oncology Biometrics, Oncology R&D, AstraZeneca, Cambridge, United Kingdom; ^5^ Late Development Oncology, Oncology R&D, AstraZeneca, Gaithersburg, MD, United States; ^6^ Patient Safety Oncology, AstraZeneca, Gaithersburg, MD, United States

**Keywords:** immunology, biomarkers, clinical trials, methodology and modeling, immunotherapy, computational methods, biostatistics, lung cancer

## Abstract

**Purpose:**

Immune-mediated adverse events (imAEs) may be associated with response to immune checkpoint inhibitors. We assessed the relationship between imAE development and efficacy in metastatic non-small-cell lung cancer patients treated with durvalumab (anti-programmed cell death ligand-1 [PD-L1]) alone or in combination with tremelimumab (anti-cytotoxic T-lymphocyte-associated protein 4).

**Methods:**

The analysis used individual patient-level data from 307 and 310 patients in the monotherapy and combination arms of MYSTIC, respectively. We evaluated the association between treatment efficacy and development of imAEs using univariate and multivariate survival analyses. Using machine learning, we built a predictive model utilizing baseline clinical and laboratory features to identify patients at risk of developing imAEs and further evaluated patient survival based on a threshold index extracted from the model.

**Results:**

Patients who developed any grade of imAE had improved overall survival versus patients without (hazard ratio [HR] 0.51; 95% confidence interval [CI]: 0.41–0.62). imAE development was associated with improved overall survival (HR 0.54; 95% CI 0.44–0.66) in a multivariate Cox proportional hazard model considering patient demographic features and baseline characteristics. Higher odds of imAE development were observed (odds ratio 3.023; 95% CI: 1.56–5.83) in responders versus non-responders in patients treated with immunotherapy. Based on baseline characteristics, the random forest classification algorithm was used to formulate a predictive model to identify patients at increased risk of developing imAEs during treatment.

**Conclusion:**

*Post-hoc* exploratory analysis found that the efficacy of immunotherapy was improved in patients who developed on-treatment imAEs. This was independent of severity of imAEs or the need for steroid treatment, which is important in allowing patients to remain on treatment and derive optimal clinical benefit. Further research is warranted to establish the correlation between incidence of imAEs and efficacy in this patient population.

## Introduction

Immune checkpoint inhibition (ICI) has revolutionized the treatment of cancer over the last decade, with multiple checkpoint inhibitors approved in solid tumors as well as for some hematologic malignancies (1, 2). The first approved checkpoint inhibitor was ipilimumab, an anti-cytotoxic T-lymphocyte-associated protein 4 (CTLA-4) monoclonal antibody (3). Subsequently, antibodies targeting programmed cell death-1 (PD-1; nivolumab, pembrolizumab, and cemiplimab) (4–6) or its ligand, programmed cell death ligand-1 (PD-L1; atezolizumab, avelumab, and durvalumab) (7–9) have also gained approvals and become integrated into the standard-of-care in many tumor types.

Despite significant clinical benefits, the use of immunotherapy can result in immune-mediated toxicities in up to 85% of patients, although this varies by agent and across tumor types (10). Such toxicities are commonly gastrointestinal, respiratory, endocrine, or dermatologic in nature (11). Mechanistically, this toxicity is believed to be caused by aberrant activation of autoreactive T or B cells (12), inhibition of regulatory T cells (13), and/or activation of tumor-reactive T cells that share an antigen with normal tissue (14). Frontline management includes withholding immunotherapy and starting corticosteroids (15). If response is inadequate, second-line immunosuppressive agents such as infliximab, vedolizumab, mycophenolate, and azathioprine are recommended (15). To date, there are no reliable tools to predict which patients will develop immune-mediated adverse events (imAEs) during treatment with immunotherapy. Prior studies have shown that clonal expansion of cytotoxic CD8 T cells precedes ipilimumab-related toxicity (16) and that patients with tumors characterized by a high mutational burden are at increased risk of immune-mediated toxicity (17). While these analyses investigated only single features, the role of multiple features in relation to the development of imAEs was evaluated in a proof-of concept study, which used real-world, reported adverse event (AE) data and molecular genomics to identify a bivariate regression model of lymphocyte cytosolic protein 1 (LCP1) and adenosine diphosphate dependent glucokinase (ADPGK) that could accurately predict imAEs (18). However, there remains a need for a comprehensive approach to identify patients who might experience imAEs during treatment using baseline information.

Several retrospective studies have shown an association between the development of imAEs and both treatment response and survival. This leads us to consider the fact that widespread systemic immune system activation and auto-reactivity may be simultaneously reflective of antitumor response. In a retrospective analysis combining seven trials in patients with metastatic or locally advanced urothelial carcinoma, overall survival (OS) was longer in patients with imAEs versus those without imAEs (hazard ratio [HR], 0.45; 95% confidence interval [CI], 0.39–0.52) (19). Similarly, incidence of imAEs was associated with longer relapse-free survival in patients with stage III melanoma who received pembrolizumab (HR, 0.61; 95% CI, 0.39–0.95; P = 0.03) (20).

Durvalumab is a human immunoglobulin G1 kappa monoclonal antibody that inhibits PD-L1 binding to PD-1 and CD80; tremelimumab is a monoclonal immunoglobulin G2 antibody binding to CTLA-4 which, in combination with durvalumab, has shown clinical efficacy in patients with advanced non-small-cell lung cancer (NSCLC) (7, 21). The phase III MYSTIC trial compared durvalumab alone or in combination with tremelimumab versus standard of care (SOC) chemotherapy in treatment-naïve patients with metastatic NSCLC whose tumors expressed PD-L1 in ≥25% of tumor cells (TC ≥ 25) (22). In the MYSTIC trial, durvalumab alone or in combination with tremelimumab did not meet statistical significance for OS versus SOC chemotherapy. However, a clinically meaningful improvement was observed in the durvalumab monotherapy group (HR 0.76; 97.54% CI: 0.56–1.02; *p* = 0.036) suggesting that some patients in MYSTIC did derive benefit from durvalumab monotherapy. Here, we report results from an analysis of MYSTIC patient data designed to better understand the effect of imAEs on treatment efficacy, allowing the development of a predictive model to identify patients with a high probability of experiencing on-treatment immune-mediated toxicity.

## Materials and methods

### Patients

Full eligibility criteria for the MYSTIC clinical trial (NCT02453282) have been previously reported (22). In brief, adults with stage IV NSCLC were eligible provided they had not previously received systemic therapy for advanced or metastatic NSCLC, had an Eastern Cooperative Oncology Group (ECOG) performance status of 0 or 1, demonstrated measurable disease according to Response Evaluation Criteria in Solid Tumors (RECIST) version 1.1 (23), and had known tumor PD-L1 expression status prior to randomization. Patients with sensitizing epidermal growth factor receptor (EGFR) or anaplastic lymphoma kinase (ALK) genetic alterations and those with symptomatic, unstable brain metastases were excluded. With the exception of vitiligo or alopecia and hypothyroidism, patients with active or prior documented autoimmune or inflammatory disorders were also excluded from the study.

The MYSTIC study was performed in accordance with the Declaration of Helsinki and the International Conference on Harmonization Good Clinical Practice Guidelines. The protocol and all modifications were approved by the institutional review boards or ethics committees of all participating centers and the relevant regulatory authorities. All participants provided written informed consent.

### Patient and public involvement statement

This study utilized patient data from the MYSTIC trial to help develop a predictive model that could identify patients at a high risk of experiencing on-treatment immune-mediated toxicity during the course of immunotherapy. Patient response and safety data were analyzed retrospectively.

### Study design and treatment

Patients were randomized in a 1:1:1 ratio in a stratified manner according to PD-L1 expression (25% cutoff) and histology (squamous vs non-squamous) to receive durvalumab plus tremelimumab, durvalumab monotherapy or SOC chemotherapy (**Supplemental Figure S1**). Patients in the durvalumab plus tremelimumab group received durvalumab 20 mg/kg and tremelimumab 1 mg/kg *via* intravenous (IV) infusion every 4 weeks (q4w) for up to four doses/cycles and then continued with durvalumab 20 mg/kg q4w from week 16 until disease progression. Patients in the durvalumab monotherapy group received durvalumab 20 mg/kg *via* IV infusion q4w. Patients in the chemotherapy arm received 4–6 cycles of platinum-based doublet chemotherapy of the investigator’s choice.

### Endpoints

The primary endpoints were OS (time from randomization to death due to any cause) for both immunotherapy arms versus chemotherapy and progression-free survival (PFS; time from randomization to objective disease progression according to blinded independent central review, or death) for durvalumab plus tremelimumab versus chemotherapy, all assessed in patients with ≥25% of TCs expressing PD-L1 (PD-L1 TC ≥25%). Secondary endpoints included PFS for durvalumab versus chemotherapy, and objective response rate and duration of response (DOR) for both immunotherapy arms versus chemotherapy (all in patients with PD-L1 TC ≥25%), as assessed using RECIST version 1.1, as well as safety and tolerability (22).

### Assessment

During medical review of the MYSTIC trial, an AE consistent with an immune-mediated mechanism of action with no clear alternate etiology was adjudicated as an imAE by the sponsor. Serologic, immunologic, and histologic (biopsy) data, as appropriate, were used to support characterization of an imAE. Unless otherwise indicated, all data regarding imAEs were adjudicated. The severity of imAEs was graded according to the National Cancer Institute’s Common Terminology Criteria for Adverse Events (CTCAE version 4.03).

### Statistical analysis

Kaplan–Meier analysis was used to compare patient survival outcomes (OS, PFS) based on development of imAEs in the immunotherapy arms, irrespective of PD-L1 expression and (separately) in subgroups based on PD-L1 expression (cut-off, 25%). Since imAE is an on-treatment phenomenon, landmark analysis based on imAE median time to onset was also used to account for immortal time bias. Treatment efficacy was compared between patients experiencing high-grade (grade ≥3) imAEs and patients experiencing low-grade (grade ≤2) imAEs using Kaplan–Meier analysis, as well as between the low-grade imAE patient cohort and the no imAE patient cohort. We also explored the effect of steroid use on treatment efficacy in patients experiencing imAEs using Kaplan–Meier analysis. Finally, we used the Kaplan–Meier method to compare the survival benefit for patients with imAEs in the immunotherapy arms over patients in the chemotherapy arm, employing a Restricted Mean Survival Time (RMST) analysis to overcome the violation of proportional hazards assumption (as observed in early crossing over of Kaplan–Meier curves) (24).

Demographic features (age, weight, sex, and race) and potential prognostic factors or predictive biomarkers of efficacy (baseline ECOG performance status, histology, presence of liver metastasis, PD-L1 expression, and tumor mutational burden) were considered in addition to incidence of imAEs in a step-wise multivariate Cox regression model to account for any possible confounding effect. All covariates were initially considered for selection. The significance levels for entry and for stay were set at 0.25 (suggested to be set conservatively at ≥0.15) (25). The best candidate final regression model was then identified by eliminating the covariates with a *p*-value of >0.05 one at a time until all regression coefficients were significantly different from 0 at the chosen alpha level of 0.05.

The association between objective response and development of imAEs was explored using a binary outcome logistic regression model. To examine the effect of exposure duration (difference in days between the treatment stop and start dates) on the development of imAEs, responder status (Yes/No; defined as those patients who achieved either a confirmed complete response [CR] or partial response [PR]), duration of exposure, and a multiplicative interaction term were included as covariates in the model.

### Machine learning classification

A predictive model was developed using the random forest algorithm (26) to identify patients at risk of experiencing immune-mediated toxicities. Baseline demographic features, potential prognostic factors and predictive markers of efficacy, and several laboratory parameters were used in model building (**Supplemental Table S1**). The 10 most significant features in this classification problem were noted using random forest feature selection. Both “accuracy” and “Gini” importance measures were taken into consideration for feature selection (26). A simpler predictive model was then developed using only these 10 features to reduce the complexity of the original model. A baseline threshold immune toxicity index was extracted (random forest model classification cut-off) from the model to identify patients more likely to experience imAEs during the course of immunotherapy. Patient survival was evaluated based on the model-informed baseline threshold immune toxicity index. An out-of-bag (OOB) error estimate (26) was used to measure the predictive ability of the model. In random forest, cross-validation on a separate dataset is not required to obtain an unbiased estimate of prediction error (26). OOB error is estimated internally using approximately one-third of cases excluded from the bootstrap sample. All analyses were performed using R statistical software.

## Results

Data from 902 patients (307 randomized to monotherapy, 310 to combination therapy, and 285 to chemotherapy) who participated in the trial were included in the analysis. Due to restrictions on secondary usage of data, the number of patients in each treatment arm in our analysis differs from the actual number of patients in the MYSTIC trial (374 randomized to monotherapy, 372 to combination therapy, and 372 to chemotherapy) (22). Patient demographics and baseline characteristics of these 902 patients were well balanced across treatment arms (**Supplemental Table S2**).

**Figure 1:**
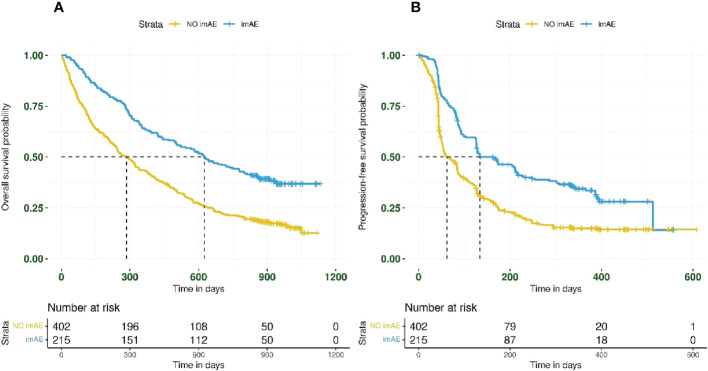
Kaplan–Meier plots for **(A)** overall survival and **(B)** progression-free survival by imAE development in patients treated with immunotherapy (*n* = 617). Data from patients randomized to durvalumab (*n* = 307) were combined with those from patients randomized to durvalumab plus tremelimumab (*n* = 310). imAE, immune-mediated adverse event.

**Figure 2:**
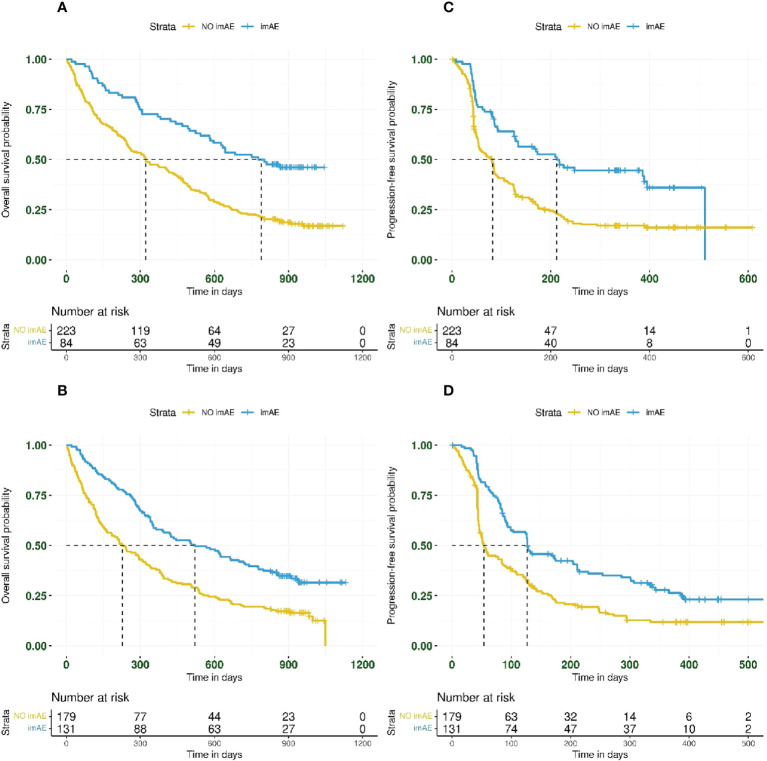
Kaplan–Meier plots for overall survival and progression-free survival based on imAE development in the durvalumab arm (panels **A**, **C** for OS and PFS, respectively) and the durvalumab plus tremelimumab arm (panels **B**, **D** for OS and PFS, respectively). imAE, immune-mediated adverse event; OS, overall survival; PFS, progression-free survival.

imAEs were observed in 215/617 patients (35%) from the immunotherapy arms, including 84/307 patients (27%) from the durvalumab arm and 131/310 patients (42%) from the durvalumab plus tremelimumab arm. In univariate analysis of both immunotherapy arms combined and irrespective of PD-L1 status, patients with imAEs had improved OS (HR 0.51; 95% CI: 0.41–0.62) (**Figure 1A**) and PFS (HR 0.54; 95% CI: 0.44–0.66) (**Figure 1B**), compared with patients without imAEs. Median time to onset of imAES was 34 days and the landmark analysis accounting for immortal time bias, which excluded patients who died (OS) or progressed (PFS) before Day 34, also reported improved OS (HR 0.55; 95% CI: 0.44–0.67) and PFS (HR 0.60; 95% CI: 0.49–0.74) in patients who had imAEs versus those who did not (**Supplemental Figure S2**). We also assessed survival benefit associated with incidence of imAEs in each immunotherapy arm separately. A similar improvement in treatment efficacy was seen in each arm for patients with imAEs versus patients without immune-mediated toxicity both for OS (durvalumab arm: HR 0.44; 95% CI: 0.31–0.61 and durvalumab plus tremelimumab arm: HR 0.52; 95% CI: 0.40–0.68) and for PFS (durvalumab arm: HR 0.49; 95% CI: 0.36–0.68 and durvalumab plus tremelimumab arm: HR 0.54; 95% CI: 0.42–0.71) (**Figure 2**). Furthermore, when considering only patients with imAEs, there was no significant difference in OS between the two immunotherapy arms, with HR 0.70 (95% CI: 0.49–1.01) for patients in the durvalumab arm versus those in the combination arm (**Supplemental Figure S3**). Therefore, patients in both immunotherapy arms were grouped together for subsequent analyses.

**Figure 3:**
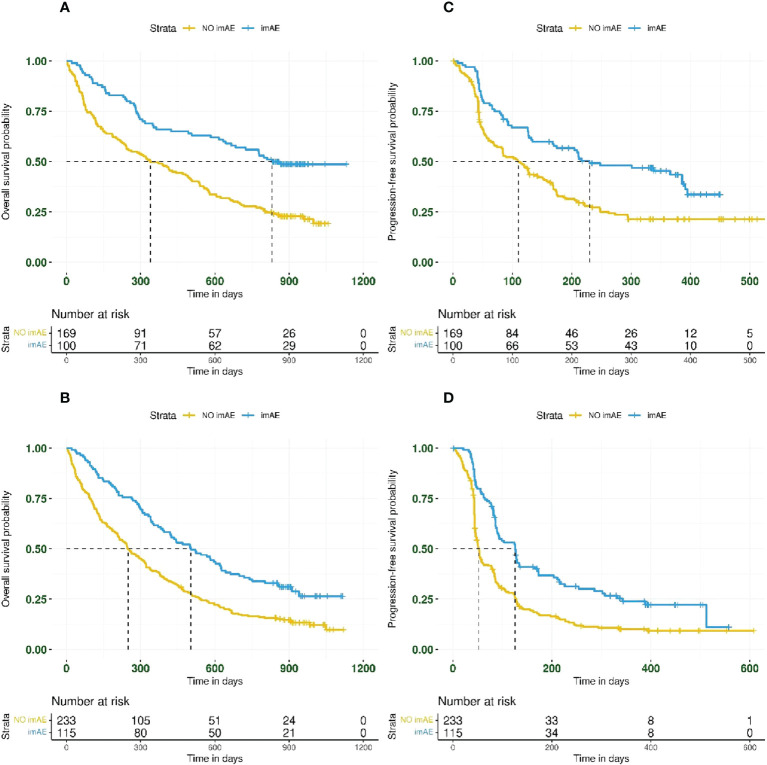
Kaplan–Meier plots for overall survival and progression-free survival based on imAE development in patients with PD-L1 TC ≥25% (panels **A**, **C** for OS and PFS, respectively) and in patients with PD-L1 TC <25% (panels **B**, **D** for OS and PFS, respectively). Data from patients randomized to durvalumab (n = 307) were combined with those from patients randomized to durvalumab plus tremelimumab (n = 310) before analyzing by PD-L1 subgroup. imAE, immune-mediated adverse event; OS, overall survival; PD-L1, programmed cell death ligand-1; PFS, progression-free survival; TC, tumor cell.

In total, 269/617 patients (44%) treated with immunotherapy had PD-L1 TC ≥25%. The incidence of any-grade imAEs was 37% (100/269 patients) in patients with PD-L1 TC ≥25% and 33% (115/348 patients) in patients with PD-L1 <25%. The association between immune-mediated toxicity and improved OS and PFS was observed regardless of PD-L1 expression (**Figure 3**). For OS and PFS, respectively, the HRs for patients with imAEs versus those lacking imAEs were 0.46 (95% CI: 0.33–0.64) and 0.55 (95% CI: 0.40–0.76) in the PD-L1 TC ≥25% subgroup (**Figures 3A, C**) and 0.55 (95% CI: 0.42–0.71) and 0.53 (95% CI: 0.41–0.68) in the PD-L1 TC <25% subgroup (**Figures 3B, D**).

As previously reported, neither durvalumab alone nor durvalumab plus tremelimumab improved survival outcomes compared with chemotherapy in the MYSTIC trial in patients with PD-L1 TC ≥25% (22). In the present analysis, although median OS was longer in patients with imAEs treated with immunotherapy than in those receiving chemotherapy, the Kaplan–Meier curves for these two patient groups crossed over at an early timepoint, thus violating the proportional hazards assumption (**Supplemental Figure S4**). An RMST analysis of OS also showed improved treatment efficacy in patients with imAEs from the immunotherapy arms compared with patients treated with chemotherapy (**Supplemental Figure S5** and **Supplemental Table S3**).

The baseline covariates listed in **Supplemental Table S2** were all considered in a multivariate analysis to further assess the relationship between treatment efficacy and incidence of imAEs. Development of imAEs was associated with improved OS (HR 0.54; 95% CI: 0.44–0.66) in the multivariate Cox regression model (where covariates were chosen in the final model by a stepwise variable selection procedure), with a similar outcome for PFS (HR 0.57; 95% CI: 0.47–0.70) (**Table 1**).

**Table 1:** Multivariate analysis for overall survival and progression-free survival in the immunotherapy arms combined.

Covariate	HR	95% CI
**Overall survival**		
imAE (Yes vs No^a^)	0.54	(0.44–0.66)
ECOG performance status (≥1 vs 0^a^)	1.55	(1.28–1.89)
PD-L1 (TC ≥25% vs TC <25%^a^)	0.71	(0.59–0.86)
Histology (Squamous vs Non-squamous^a^)	1.36	(1.11–1.65)
Liver metastasis (Yes vs No^a^)	1.27	(1.01–1.60)
TMB (≥20 mut/Mb vs <20 mut/Mb^a^)	0.66	(0.51–0.86)
**Progression-free survival**		
imAE (Yes vs No^a^)	0.57	(0.47–0.70)
ECOG performance status (≥1 vs 0^a^)	1.34	(1.10–1.62)
PD-L1 (TC ≥25% vs TC <25%^a^)	0.63	(0.52–0.77)
Liver metastasis (Yes vs No^a^)	1.29	(1.02–1.62)
TMB (≥20 mut/Mb vs <20 mut/Mb^a^)	0.68	(0.53–0.89)

CI, confidence interval; ECOG, Eastern Cooperative Oncology Group; HR, hazard ratio; imAE, immune-mediated adverse event; mut/Mb, mutations/megabase; PD-L1, programmed cell death ligand-1; TC, tumor cell; TMB, tumor mutational burden.

imAE development was assessed along with other significant covariates chosen by the stepwise multivariate Cox regression model.

^a^Indicates reference level. For example, HR = 0.54 favors imAE = Yes.

Of 215 patients experiencing imAEs in the immunotherapy arms, 173 patients (80%) had low-grade (grade ≤2) imAEs, including 68/84 patients (81%) in the durvalumab arm and 105/131 patients (80%) in the durvalumab plus tremelimumab arm. OS and PFS did not differ between patients with high-grade versus low-grade imAEs (**Supplemental Figure S6**). However, OS and PFS were both improved (**Supplemental Figure S7**) in patients with only low-grade imAEs (both immunotherapy arms grouped together) compared with patients who didn’t experience any imAEs (OS: HR 0.50; 95% CI: 0.40–0.62; PFS: HR 0.56; 95% CI: 0.45–0.69).

**Figure 4:**
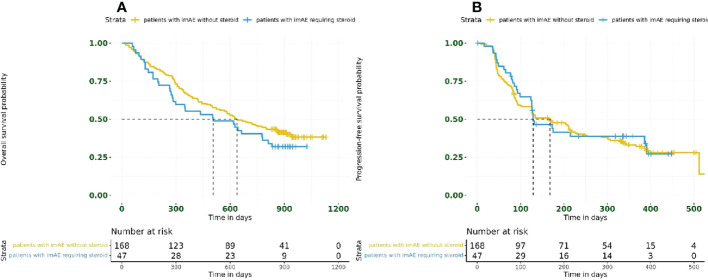
Kaplan–Meier plots for **(A)** overall survival and **(B)** progression-free survival based on steroid usage in patients with imAEs from the immunotherapy arms combined (*n* = 215). imAE, immune-mediated adverse event.

Overall, 47/215 patients (22%) had immune-mediated toxicity requiring steroid use. Survival outcomes were unaffected by steroid use in patients with imAEs; for patients receiving steroids, the HR for OS was 1.27 (95% CI: 0.85–1.89) and the HR for PFS was 0.93 (95% CI: 0.62–1.41) compared with those patients who did not require steroid treatment (**Figure 4**).

Among the 617 patients treated with immunotherapy included in our analysis, 152 patients (25%) had an objective response (CR or PR). The incidence of imAEs was higher in responders (79/152 patients; 52% [95% CI: 44–59]) than in non-responders (136/465 patients; 29% [95% CI: 25–33]) (**Table 2**). The association between development of imAEs and objective response was evaluated through a logistic regression model adjusted for the duration of exposure. Higher odds of imAE development were observed (odds ratio 3.023; 95% CI: 1.56–5.83) in responders versus non-responders (**Table 2**). The interaction term between response and duration of exposure was significant in the model, indicating that responders and non-responders had a differential proclivity for development of imAEs given the same duration of exposure.

**Table 2:** Relationship between imAE development and objective response.

Odds of imAE development				
	OR	95% CI		*p*-value
Response (Yes/No)^a^	3.023	1.56–5.83		0.0009
**Interaction between response and imAE development**				
	**Coefficient**	**SE**		***p*-value**
Response (Yes/No) ^a^	1.10	0.33		***
Exposure duration	0.002	0.0005		***
Interaction (response x exposure duration)	–0.001	0.0007		*
**Patients with imAE stratified by response**				
**Patients treated with immunotherapy**	**Responders (*n* = 152)**		**Non-responders (*n* = 465)**	
Incidence of imAEs: *n* (%)	79 (52)		136 (29)	
95% CI	44–59		25–33	

CI, confidence interval; imAE, immune-mediated adverse event; OR, odds ratio; SE, standard error.

^a^Responder status “No” is the reference level.

OR calculated after adjusting for duration of exposure.

Responders have higher odds of developing imAEs after adjusting for duration of exposure. A significant interaction term indicates different inclination towards imAE development among responders versus non-responders, given the same duration of exposure.

p-value significance: *, p ≤ 0.05; ***, p ≤ 0.001.

Using patient baseline characteristics (**Supplemental Table S1**), a random forest predictive model (R script; **Supplementary File**) was developed to identify patients with a higher likelihood of experiencing imAEs during treatment with immunotherapy. Patients with immune toxicity index above the reference level of 0.5 (≥0.5 vs <0.5) were classified (OOB error estimate 12.28%) as patients who would experience imAEs during the course of immunotherapy. The top 10 features associated with imAE development were identified (**Supplemental Figure S8**) from the entire feature-space (**Supplemental Table S1**) using random forest feature selection (26). The simpler model, developed using these 10 features, was able to classify patients with imAEs with an OOB error estimate of 14.3% (**Supplemental Figure S9**). The model-informed immune toxicity index was then assessed with other baseline covariates from **Supplemental Table S2** in a multivariate Cox regression model for treatment efficacy. Based on this analysis, immune toxicity index ≥0.5 versus <0.5 was associated with improved OS (HR 0.57; 95% CI: 0.46–0.71), with a similar outcome for PFS (HR 0.60; 95% CI: 0.48–0.74) (**Supplemental Table S4**).

## Discussion

### Association between imAEs and clinical outcome

The primary analysis of the MYSTIC trial found no statistically significant difference in OS between immunotherapy (either durvalumab or durvalumab plus tremelimumab) and chemotherapy (22). The aim of this retrospective analysis was to improve our understanding of the survival benefit associated with imAE development in the immunotherapy arms of the phase III MYSTIC trial. Using both univariate and multivariate analysis, we found that both OS and PFS tended to be improved in patients who experienced imAEs on immunotherapy when compared with those who did not develop imAEs. Landmark analysis using imAE median time to onset (taking into account the time-dependent nature of imAE) further confirmed the survival benefit associated with imAEs – this is also in alignment with the results presented by Haratani et al. (27) Subgroup analysis showed that survival outcomes were independent of PD-L1 status (above or below a threshold of TC 25%), grade of imAE, and corticosteroid usage. Although overall rates of imAEs in MYSTIC were low, we also demonstrated that patients who experienced imAEs during immunotherapy had noticeably longer OS than patients randomized to SOC chemotherapy. Substantial association between development of imAEs and objective response was seen in patients treated with immunotherapy in a multivariate analysis adjusted for duration of exposure. The significant interaction term between response and duration of exposure in the logistic regression model suggests that, given the same duration of exposure, immunotherapy responders and non-responders have different likelihoods of developing imAEs. However, these analyses were limited by their retrospective and exploratory nature.

Survival benefit associated with incidence of imAEs has been previously reported in the literature across various tumor types; however, most had relatively small cohorts and significant limitations adjusting for confounders. However, recent examples of larger-scale analyses reflect the findings reported here. For example, one such analysis looked at pooled safety and efficacy data from 1783 patients with various solid tumors, including NSCLC, treated with avelumab in the JAVELIN Solid Tumor and JAVELIN Merkel 200 trials (28). Patients experiencing imAEs (overall incidence 16.5%), had a greater improvement in OS than those who did not. In exploratory analyses including 1747 patients with urothelial cancer who received a PD-1/PD-L1 inhibitor across seven clinical trials, patients experiencing on-treatment imAEs also demonstrated improved OS versus those with no imAEs (19). We also highlight that much of the current literature has considered only a single immunotherapy mode-of-action, thus our analysis of imAEs in patients receiving a combination of PD-(L)1 and CTLA-4 agents is of particular note.

The exact mechanism for the association between development of imAEs and ICI-triggered response is not entirely clear. It is postulated that there is a shared antigen between the tumor and otherwise normal surrounding tissue from which the tumor arises, with toxicity a byproduct of T-cell stimulation and activation at the site of the tumor. Several clinical observations support this notion, including an increase in response for patients with metastatic melanoma who develop vitiligo after treatment with immunotherapy (14, 20), the association between acute interstitial nephritis and clinical response in renal cell carcinoma (29), and the high incidence of pneumonitis in patients with NSCLC receiving immune checkpoint inhibitors (30). It has also been shown that there is an association between tumor-tissue similarity and frequency of autoimmune toxic effects on a molecular level. Using advanced sequencing techniques, researchers demonstrated that several T-cell clonotypes present in NSCLC tumors were also present at the site of skin toxicity (31), providing evidence to support the theory that shared antigenicity in two separate organ systems is one potential mechanism for the observed toxicity.

An important unanswered clinical question is the effect of glucocorticoid use on immunotherapy efficacy. While it has been shown through *in vivo* studies that corticosteroids inhibit effector T-cell proliferation and promote regulatory T-cell expansion (32), it is unclear how this may affect efficacy in the context of imAEs. This is particularly important given the need for effective management of imAEs to ensure that patients can remain on treatment and derive optimal clinical benefit. In our analysis, the improvement in PFS and OS related to imAEs was independent of steroid exposure. This is consistent with data presented in the context of urothelial cancer, which showed that use of corticosteroids did not negatively affect either the chance of developing a response to PD-1/PD-L1 inhibitors or the DOR (19). Analysis of data from patients with NSCLC is also in alignment, reporting that receipt of corticosteroids for imAEs did not affect OS (33). Our ability to make definitive conclusions based on the dataset presented is limited due to a small sample size, with only 47 patients receiving immunotherapy requiring steroid administration. Future, prospective, and biological studies are warranted to better address this question.

### Developing a predictive tool for imAEs

We developed a machine learning predictive model to identify patients who might experience imAEs during treatment with immunotherapy using only baseline characteristics. A model immune toxicity index was created for this purpose. We also evaluated patient survival based on this index. In our model, in which prediction was limited by the available data, patients with a higher immune toxicity index score (≥0.5; i.e. those with a higher chance of developing imAEs on immunotherapy), had a noticeable survival benefit. However, more comprehensive predictive model development using a larger database and validation using different studies is required for patient benefit.

Various baseline patient characteristics, such as differences in peripheral cytokine levels (34, 35) and differences in B-cell abundance (36), have been correlated with immune-mediated toxicity; however, these findings are preliminary and as yet there are no validated predictive biomarkers for imAE development. As immunotherapy is incorporated into earlier stages where surgical and radiotherapy treatment alone has curative potential, the risks and benefits of additional immunotherapy treatment need to be considered with a different benefit-risk perspective to that in the metastatic context. Our hope is that a comprehensive predictive model may ultimately be available to clinicians to improve risk stratification of patients and to help guide treatment decisions.

In conclusion, our analysis demonstrated improved survival outcomes in patients experiencing imAEs during immunotherapy, regardless of the severity of imAE or the need for steroid treatment, which is important in allowing patients to remain on treatment and derive optimal clinical benefit. Further analyses across different tumor types and with different combination regimens are required to investigate this relationship in greater detail. If imAE development is confirmed to be correlated with survival benefit, a comprehensive predictive model (using only baseline characteristics) that identifies patients with a greater likelihood of experiencing immune-related toxicity during treatment would significantly aid clinicians in careful monitoring and counseling of these patients.

## Data availability statement

Data underlying the findings described in this manuscript may be obtained in accordance with AstraZeneca’s data sharing policy described at https://astrazenecagrouptrials.pharmacm.com/ST/Submission/Disclosure.

## Ethics statement

Please refer to the primary publication for this trial: Rizvi et al. JAMA Oncol. 2020;6 (5):661-674. doi:10.1001/jamaoncol.2020.0237. The patients/participants provided their written informed consent to participate in this study.

## Author contributions

AD was involved in the study concept, the collection and analysis of the data, contributed to the development of the analysis plan and modeling, and was heavily involved in the writing, reviewing, and editing of the manuscript. MA was involved in the writing, reviewing, and editing of the manuscript. HMK contributed to the development of the analysis plan and was involved in reviewing and editing the manuscript. NT contributed to the development of the analysis plan and was involved in reviewing and editing the manuscript. HM contributed to the development of the analysis plan and was involved in reviewing and editing the manuscript. NS was involved in reviewing and editing the manuscript. CM contributed to the development of the analysis plan and was involved in reviewing and editing the manuscript. DZ contributed to the development of the analysis plan and was involved in the writing, reviewing, and editing of the manuscript. GMM was involved in the study concept, contributed to the development of the analysis plan, and was involved in reviewing and editing the manuscript. AD, MA, HMK, NT, HM, NS, CM, DZ, and GMM have provided consent for the analyses detailed in this manuscript to be published.

## Funding

This study was funded by AstraZeneca.

## Acknowledgments

This study was funded by AstraZeneca. Editorial support was provided by Rachel Cicchelli, PhD, of Ashfield MedComms (Macclesfield, UK), an Inizio company, and was funded by AstraZeneca.

## Conflict of interest

AD and GMM were employees of AstraZeneca at the time this work was conducted. NT, HM, NS, CM, and DZ are all employees of AstraZeneca and hold or have the option to hold stock. HMK declares receipt of institutional research grants directly to institute from Merck, Bristol-Myers Squibb, and Apexigen, personal fees outside of the work under consideration from Nektar, Immunocore, Celldex, Array Biopharma, Merck, Elevate Bio, Instil Bio, Bristol-Myers Squibb, Clinigen, Shionogi, Chemocentryx, Calithera, and Signatero, and personal fees not outside of the work under consideration from Iovance.

The remaining author declares that the research was conducted in the absence of any commercial or financial relationships that could be construed as a potential conflict of interest.

The authors declare that this study received funding from AstraZeneca. The funder of the study participated in study design, data collection, data analysis, data interpretation, and writing of the report.

## Publisher’s note

All claims expressed in this article are solely those of the authors and do not necessarily represent those of their affiliated organizations, or those of the publisher, the editors and the reviewers. Any product that may be evaluated in this article, or claim that may be made by its manufacturer, is not guaranteed or endorsed by the publisher.
