# Supplementary material for: Association between immune-mediated adverse events and efficacy in metastatic non-small-cell lung cancer patients treated with durvalumab and tremelimumab
Source: Front Immunol. 2022 Nov 3;13:1026964. doi: 10.3389/fimmu.2022.1026964 (PMC9670978; doi:10.3389/fimmu.2022.1026964)
Supplement: Supplementary file 1 [file DataSheet_1.zip › Supplementary Files_LVNG With/Dey et al_Front Immunol_Supplement.docx]

**Supplement**

**Association Between Immune-mediated Adverse Events and Efficacy in Metastatic Non-small-cell Lung Cancer Patients Treated with Durvalumab and Tremelimumab**

*Agnish Dey^1†*^, Matthew Austin^2^, Harriet M. Kluger^2^, Nataliya Trunova^3^, Helen Mann^4^, Norah Shire^5^, Claire Morgan^6^, Diansong Zhou^1^ and Ganesh M. Mugundu^1†^*

*^1^ Clinical Pharmacology and Quantitative Pharmacology, Biopharmaceuticals R&D, AstraZeneca, Boston, MA, USA, ^2^ School of Medicine, Yale University, New Haven, CT, USA, ^3^ Immuno-Oncology Franchise, Oncology R&D, AstraZeneca, Gaithersburg, MD, USA, ^4^ Oncology Biometrics, Oncology R&D, AstraZeneca, Cambridge, UK, ^5^ Late Development Oncology, Oncology R&D, AstraZeneca, Gaithersburg, MD, USA, ^6^ Patient Safety Oncology, AstraZeneca, Gaithersburg, MD, USA*

^†^AD and GMM were employees of AstraZeneca at the time this work was conducted. AD and GMM are now employed by Takeda, MA, USA.

**Supplemental Material**

**SUPPLEMENTAL TABLE S1 |** Predictive model input features.

| **Demographic features** |
| --- |
| Age |
| Sex |
| Race |
| Weight |
| **Potential prognostic factors/predictive biomarkers of efficacy** |
| ECOG performance status |
| PD-L1 expression |
| Histology |
| Liver metastasis |
| Tumor mutation burden |
| **Laboratory parameters** |
| LDH: lactate dehydrogenase |
| NLR: neutrophil to lymphocyte ratio |
| Tumor size |
| ALT: alanine transaminase |
| AST: aspartate transaminase |
| Eosinophil |
| Albumin |
| Bilirubin |
| Basophil |
| Hemoglobin |
| GGT: gamma glutamyl transferase |
| Monocyte |
| Platelet |
| Calcium |
| Sodium |
| Magnesium |
| Potassium |
| Glucose |
| Chloride |
| Creatinine |

*ECOG, Eastern Cooperative Oncology Group; PD-L1, programmed cell death ligand-1.*

*Blue: Numeric.*

*Orange: Categorical.*

**SUPPLEMENTAL TABLE S2 |** Patient demographics and baseline characteristics by treatment arm for patients included in the imAE analysis (*n* = 902).

| **Baseline characteristics, %** | **Durvalumab**  **(*n* = 307)** | **Durvalumab plus tremelimumab**  **(*n* = 310)** | **Chemotherapy**  **(*n* = 285)** |
| --- | --- | --- | --- |
| Age >65 years | 50 | 53 | 45 |
| Weight >70 kg | 44 | 45 | 42 |
| Male | 69 | 73 | 71 |
| ECOG performance status 0 | 37 | 39 | 39 |
| PD-L1 >25% | 44 | 43 | 41 |
| TMB ≥20 mut/Mb | 19 | 18 | 19 |
| Liver metastasis present | 20 | 18 | 22 |
| Squamous cell histology | 29 | 29 | 30 |
| Race: white | 60 | 62 | 59 |

*ECOG, Eastern Cooperative Oncology Group; imAE, immune-mediated adverse event; mut/Mb, mutations per megabase; PD-L1, programmed cell death ligand-1; TMB, tumor mutational burden.*

**SUPPLEMENTAL TABLE S3 |** Summary of RMST analysis (overall survival) between patients with imAEs in the immunotherapy arms combined (arm 1) and patients in the chemotherapy arm (arm 0). The ratio of RMST and RMTL between the two arms, RMST by treatment arm, and RMTL by treatment arm are shown.

|  | **Estimate** | **Lower .95** | **Upper .95** |
| --- | --- | --- | --- |
| **Between-group contrast** | | | |
| RMST ratio (arm 1)/(arm 0) | 1.038 | 0.937 | 1.151 |
| RMTL ratio (arm 1/arm 0) | 0.969 | 0.890 | 1.055 |
| **RMST by arm** | | | |
| Arm 1 RMST, days | 479.048 | 449.229 | 508.868 |
| Arm 0 RMST, days | 461.311 | 423.702 | 498.919 |
| **RMTL by arm** | | | |
| Arm 1 RMTL, days | 553.952 | 524.132 | 583.771 |
| Arm 0 RMTL, days | 571.689 | 534.081 | 609.298 |

*imAE, immune-mediated adverse event; RMST, Restricted Mean Survival Time; RMTL, Restricted Mean Time Lost.*

**SUPPLEMENTAL TABLE S4 |** Multivariate analysis for overall survival and progression-free survival in the combined immunotherapy arms assessing the predictive model ITI, baseline demographic features, and potential prognostic or predictive (bio)markers of efficacy.

| **Covariate** | **HR** | **95% CI** |
| --- | --- | --- |
| **Overall survival** | | |
| ITI (≥0.5 vs <0.5^a^) | 0.57 | (0.46–0.71) |
| ECOG performance status (≥1 vs 0^a^) | 1.46 | (1.19–1.79) |
| PD-L1 (TC ≥25% vs TC <25%^a^) | 0.74 | (0.61–0.90) |
| Histology (Squamous vs Non-squamous^a^) | 1.28 | (1.04–1.58) |
| Liver metastasis (Yes vs No^a^) | 1.32 | (1.04–1.67) |
| TMB score (≥20 mut/Mb vs <20 mut/Mb^a^) | 0.64 | (0.49–0.84) |
| Age (≥65 years vs <65 years^a^) | 0.97 | (0.80–1.18) |
| Weight (≥70 kg vs <70 kg^a^) | 0.94 | (0.76–1.17) |
| Race (White vs Non-white^a^) | 0.93 | (0.74–1.16) |
| Gender (Male vs Female^a^) | 1.19 | (0.95–1.50) |
| **Progression-free survival** | | |
| ITI (≥0.5 vs <0.5^a^) | 0.60 | (0.48–0.74) |
| ECOG performance status (≥1 vs 0^a^) | 1.23 | (1.00–1.51) |
| PD-L1 (TC ≥25% vs TC <25%^a^) | 0.63 | (0.52–0.77) |
| Histology (Squamous vs Non-squamous^a^) | 1.16 | (0.93–1.43) |
| Liver metastasis (Yes vs No^a^) | 1.35 | (1.06–1.71) |
| TMB (≥20 mut/Mb vs <20 mut/Mb^a^) | 0.67 | (0.51–0.88) |
| Age (≥65 years vs <65 years^a^) | 0.98 | (0.81–1.19) |
| Weight (≥70 kg vs <70 kg^a^) | 0.88 | (0.71–1.09) |
| Race (White vs Non-white^a^) | 0.86 | (0.68–1.07) |
| Gender (Male vs Female^a^) | 1.03 | (0.82–1.29) |

*CI, confidence interval; ECOG, Eastern Cooperative Oncology Group; HR, hazard ratio; ITI, immune toxicity index; mut/Mb, mutations per megabase; PD-L1, programmed cell death ligand-1; TC, tumor cell; TMB, tumor mutational burden.*

*^a^Indicates reference level (e.g., HR = 0.57 favors ITI ≥0.5).*

**
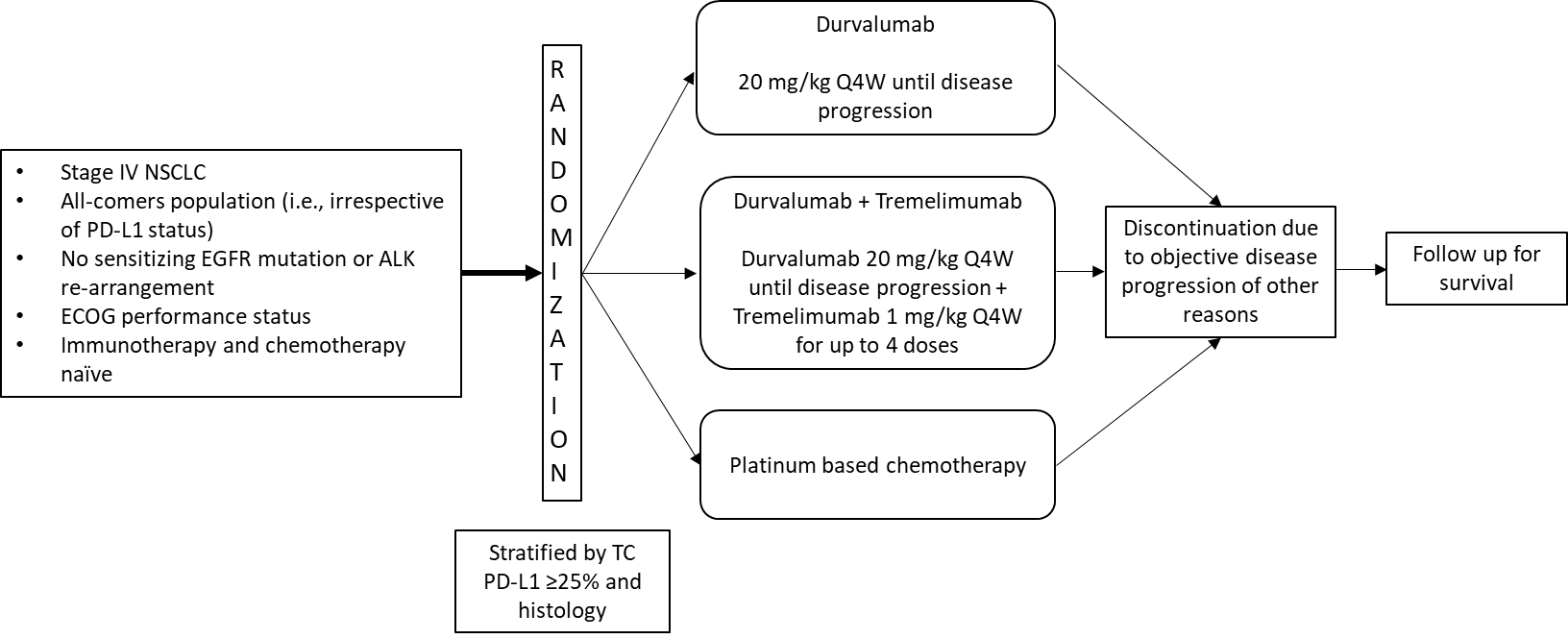
**

**SUPPLEMENTAL FIGURE S1 |** MYSTIC study design.

ALK, anaplastic lymphoma kinase; ECOG, Eastern Cooperative Oncology Group; EGFR, epidermal growth factor receptor; NSCLC, non-small-cell lung cancer; PD-L1, programmed cell death ligand-1; Q4W, every 4 weeks; TC, tumor cell.

**
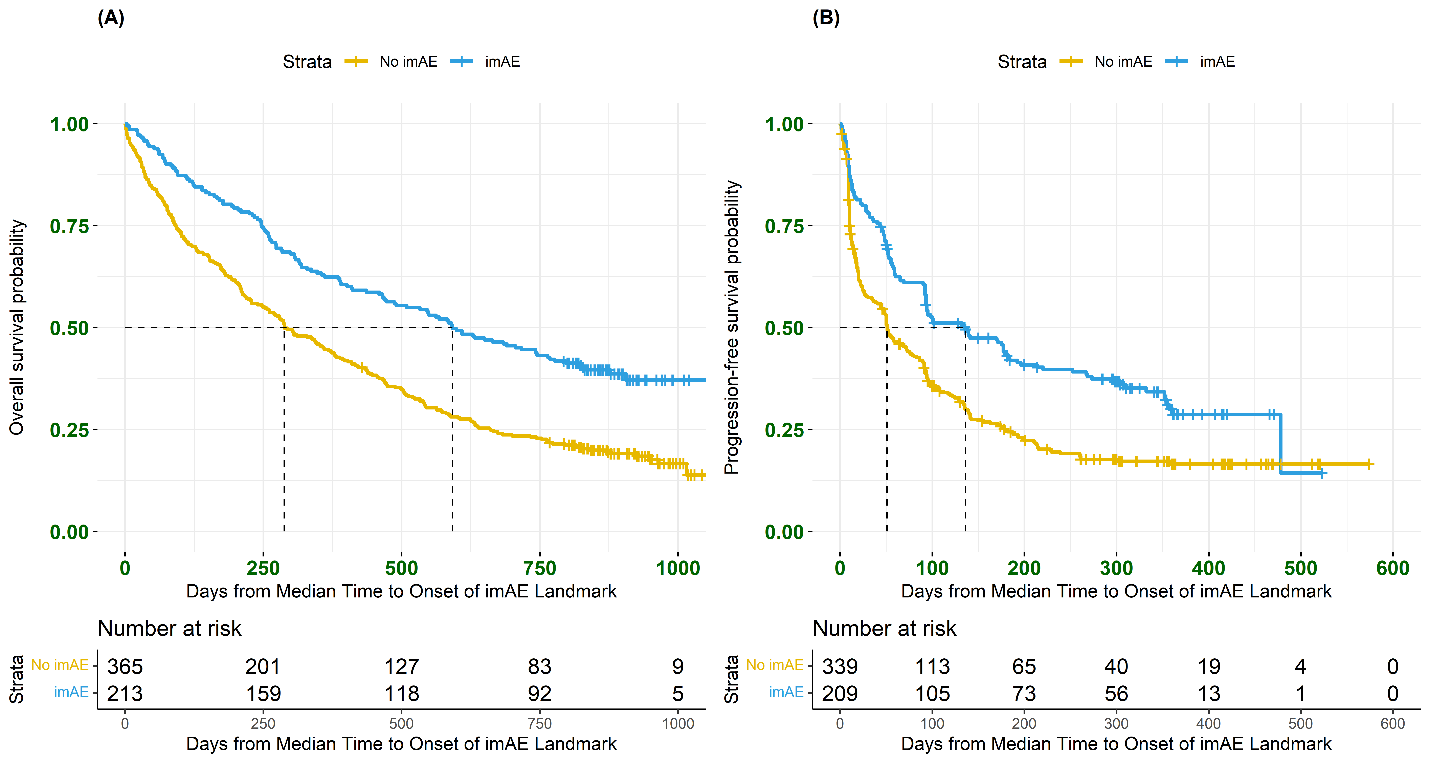
**

**SUPPLEMENTAL FIGURE S2** | Landmark (imAE median time to onset: Day 34) analysis plots for (A) overall survival and (B) progression-free survival by imAE development in patients receiving immunotherapy (*n =* 578 and *n =* 548 respectively). imAE, immune-mediated adverse event.

**
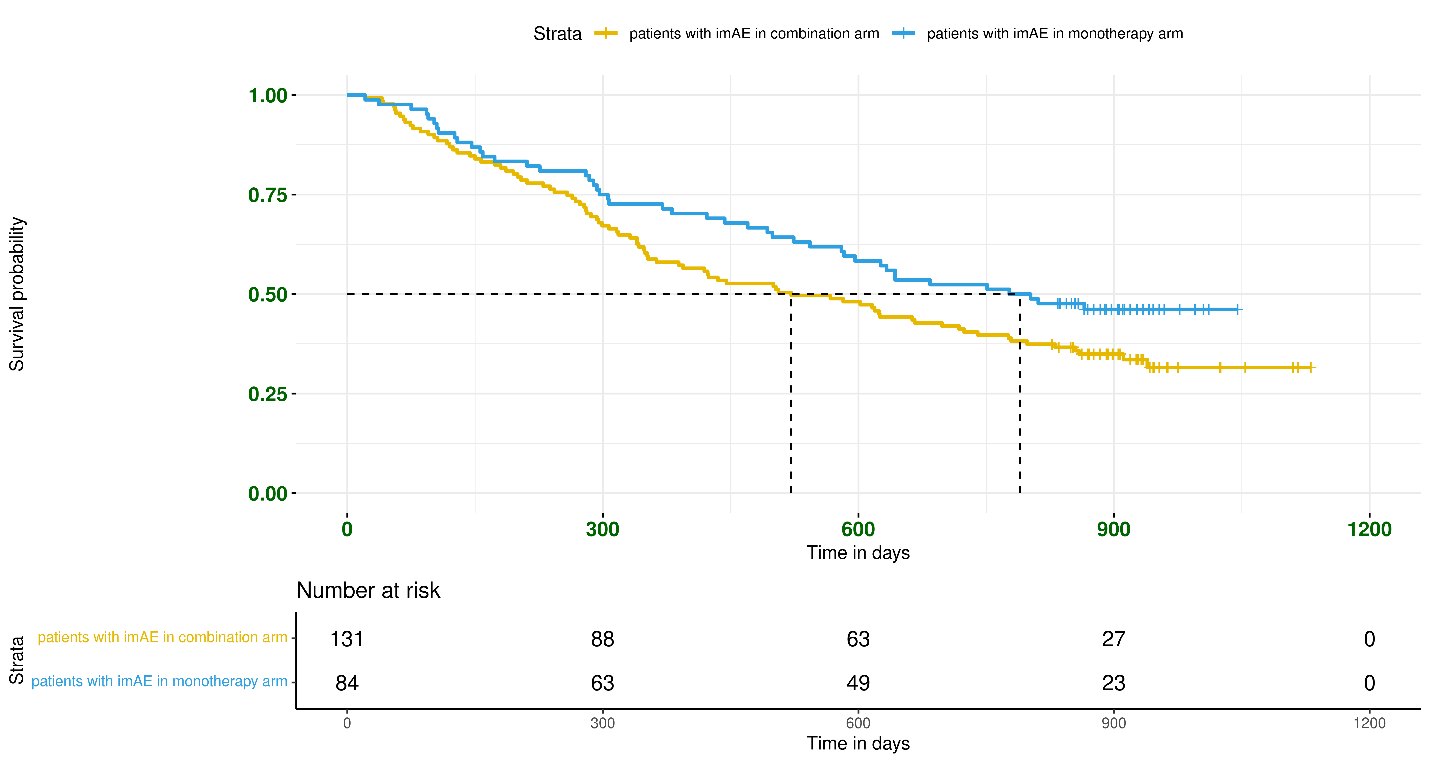
SUPPLEMENTAL FIGURE S3 |** Kaplan-Meier plot comparing overall survival for patients with imAE in the durvalumab and the durvalumab plus tremelimumab arms. imAE, immune-mediated adverse event.
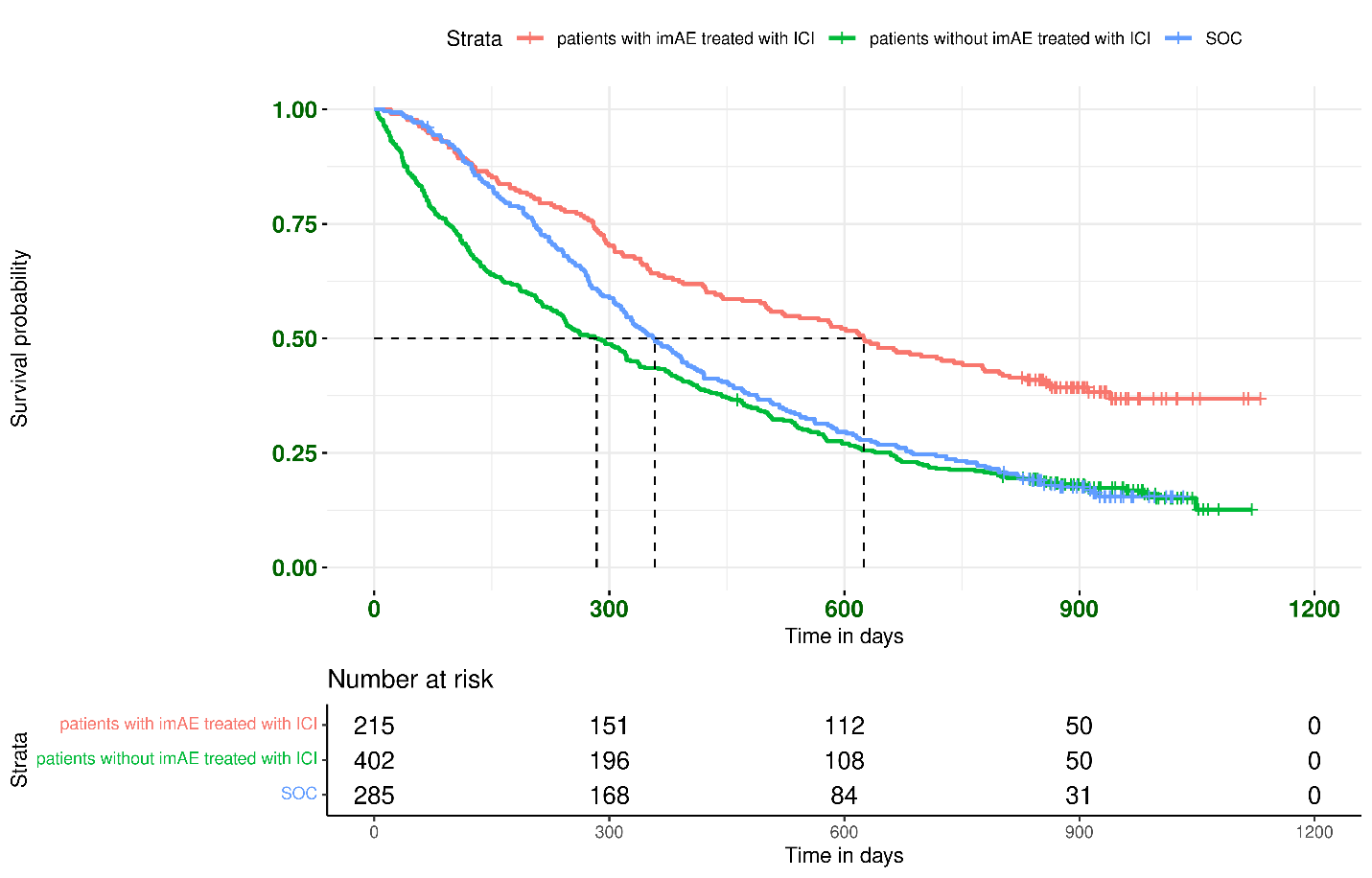
**SUPPLEMENTAL FIGURE S4 |** Overall survival comparison between patients treated with immunotherapy (data combined for the durvalumab and durvalumab plus tremelimumab arms) and with chemotherapy. Patients in the immunotherapy arms were stratified by imAE development. ICI, immune checkpoint inhibitor; imAE, immune-mediated adverse event; SOC, standard of care.


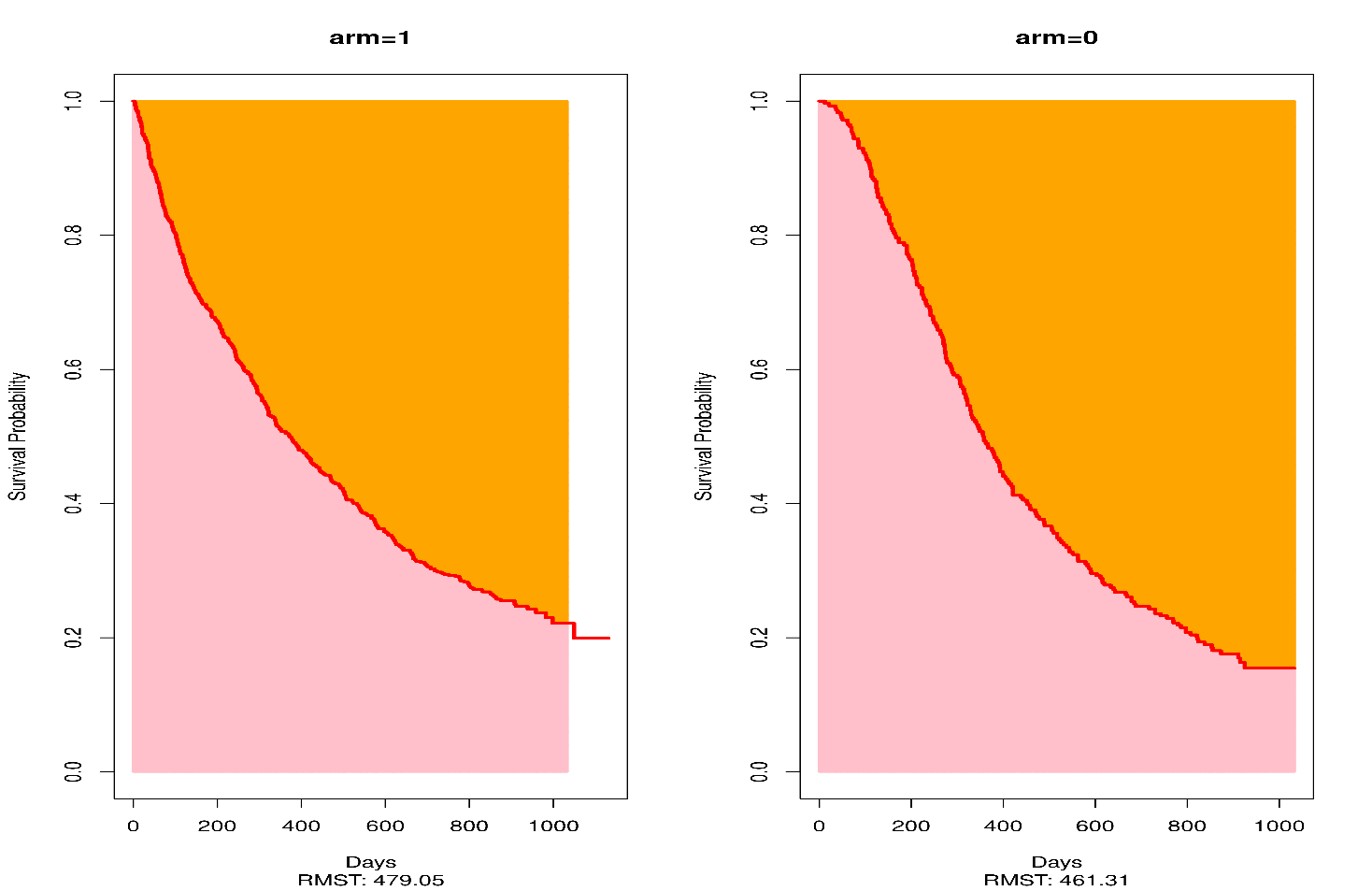
**SUPPLEMENTAL FIGURE S5 |** Restricted Mean Survival Time (RMST) analysis comparing overall survival between patients with imAEs treated with immunotherapy (arm 1: both durvalumab monotherapy and durvalumab with tremelimumab combination) and patients in the standard of care arm (arm 0). Areas highlighted in pink and orange are RMST and RMTL (Restricted Mean Time Lost) estimates, respectively. imAE, immune-mediated adverse event.


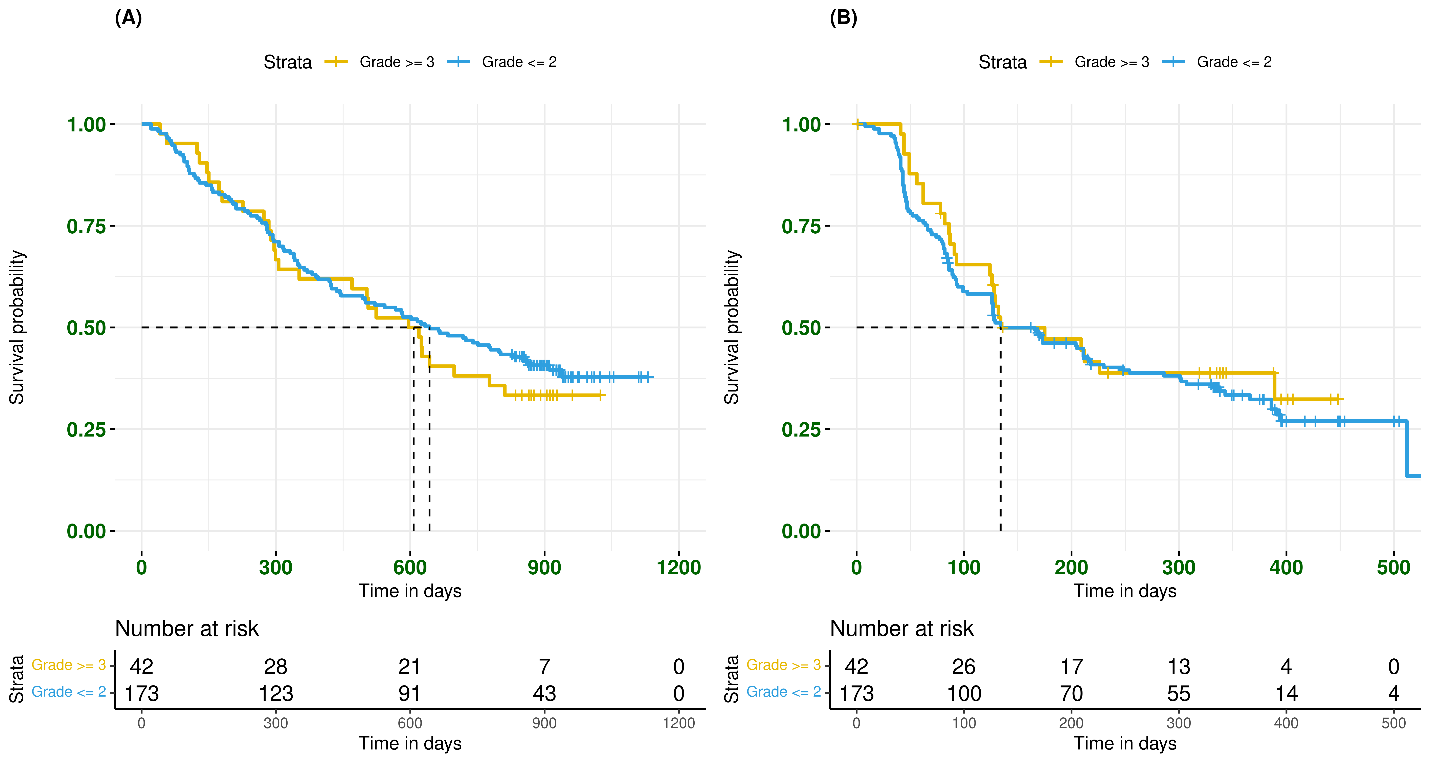
**SUPPLEMENTAL FIGURE S6 |** Kaplan-Meier plots for **(A)** overall survival and **(B)** progression-free survival by imAE grade in patients with imAEs from the immunotherapy arms combined (*n* = 215). imAE, immune-mediated adverse event.


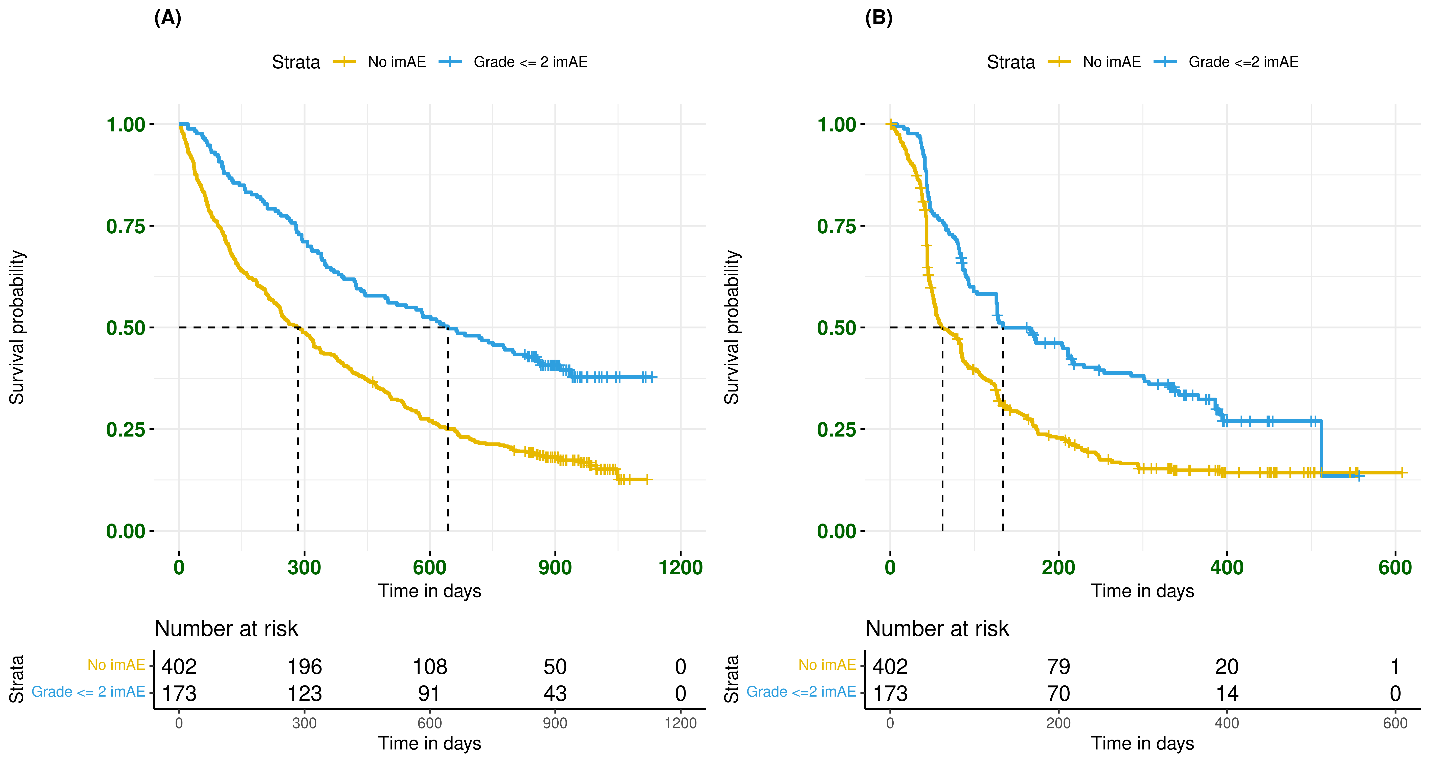
**SUPPLEMENTAL FIGURE S7 |** Kaplan-Meier plots for **(A)** overall survival and **(B)** progression-free survival comparing patients with low-grade (≤2) imAEs and patients with no imAEs from the immunotherapy arms combined (*n* = 575). Patients with high-grade (≥3) imAEs (*n* = 42) were excluded from this analysis. imAE, immune-mediated adverse event.


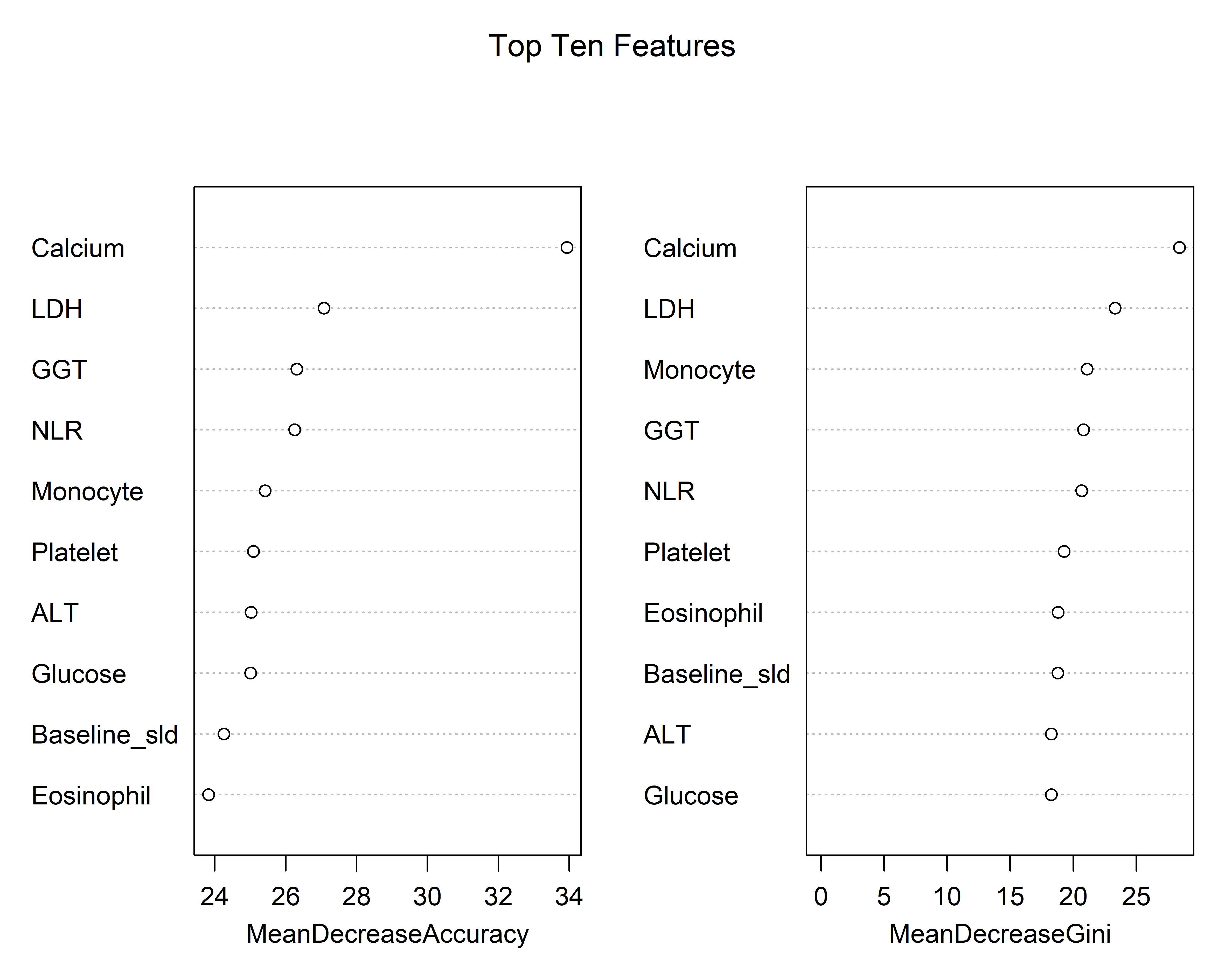


**SUPPLEMENTAL FIGURE S8 |** Top 10 features identified by the random forest feature-selection mechanism (from the entire feature space as listed in **Supplemental Table S1**). These 10 features were used to develop the final simpler predictive model. The mean decrease accuracy plot expresses how much accuracy the model loses by excluding each variable. The more the accuracy suffers, the more important the feature is for classification purpose. The mean decrease in Gini coefficient is a measure of how each variable contributes to the homogeneity of the nodes and leaves in the resulting random forest. ALT, alanine aminotransferase; Baseline_sld, baseline tumor size (sum of longest diameter); GGT, gamma glutamyl transferase; LDH, lactate dehydrogenase; NLR, neutrophil to lymphocyte ratio.


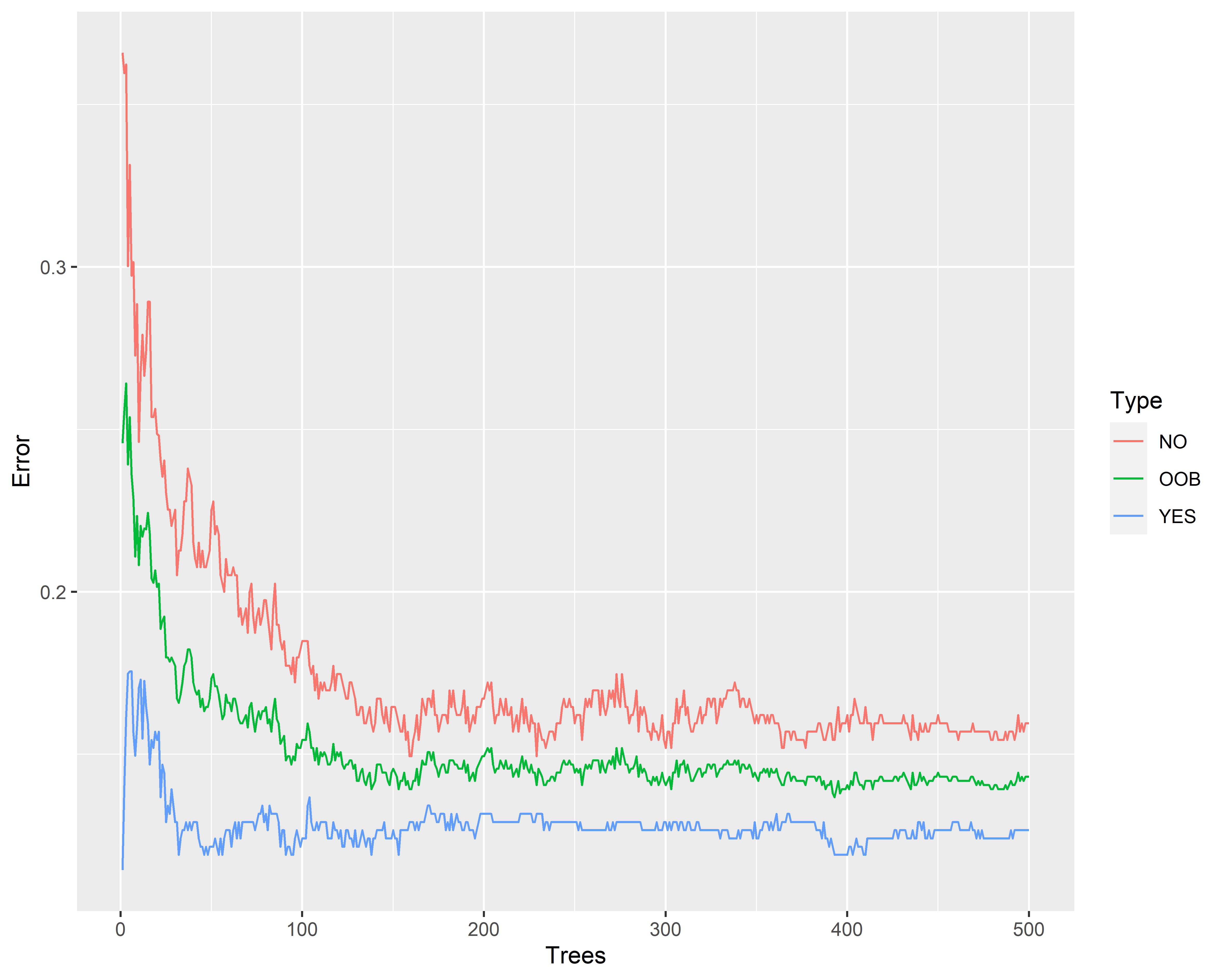


**SUPPLEMENTAL FIGURE S9 |** Out-of-bag (OOB) error estimate from the random forest classification model. The green line is the measure of overall error estimate in the two-class (imAE “YES” or “NO”) classification. imAE, immune-mediated adverse event.
