# Supplementary material for: Association between immune-mediated adverse events and efficacy in metastatic non-small-cell lung cancer patients treated with durvalumab and tremelimumab
Source: Front Immunol. 2022 Nov 3;13:1026964. doi: 10.3389/fimmu.2022.1026964 (PMC9670978; doi:10.3389/fimmu.2022.1026964)
Supplement: Supplementary file 2 [file DataSheet_2.docx]

##------Predictive Model : MYSTIC IMAE------------------------

##-------LOAD LIBRARIES----------

library(ROSE)

library(dplyr)

library(ggplot2)

library(caret)

library(randomForest)

library(vip)

##-------------------------------

##------READ DATA FILE-----------------

mys<- read.csv(file = "mystic_iolab_baseline_nov16.csv")

##----data prep for random forest--------------------------

table(mys$treatment)

## Monotherapy: 303,

## Combination: 302.

mys.io.pred.cols<- mys[,c("SUBJID", "tumor_mut",

"Gender", "Liver_met", "ECOG_group",

"pdl1_group", "histology",

"race_group", "AGE","BLWGHT", "Baseline_sld",

"CREAT_BL",

"ALB_BL", "BASO_BL", "BILI_BL", "CAL_BL",

"ALT_BL", "CHL_BL", "GGT_BL", "EOS_BL",

"GLUC_BL", "LDH_BL", "NLR_BL",

"MONO_BL", "MAG_BL", "PLAT_BL", "POT_BL",

"SOD_BL", "HEMO_BL", "AST_BL", "IMAE_Response")]

## 605 obs from 31 vars.

## Renaming lab parameters.

names(mys.io.pred.cols)[30]<- "AST"

names(mys.io.pred.cols)[29]<- "Hemoglobin"

names(mys.io.pred.cols)[28]<- "Sodium"

names(mys.io.pred.cols)[27]<- "Potassium"

names(mys.io.pred.cols)[26]<- "Platelet"

names(mys.io.pred.cols)[25]<- "Magnesium"

names(mys.io.pred.cols)[24]<- "Monocyte"

names(mys.io.pred.cols)[23]<- "NLR"

names(mys.io.pred.cols)[22]<- "LDH"

names(mys.io.pred.cols)[21]<- "Glucose"

names(mys.io.pred.cols)[20]<- "Eosinophil"

names(mys.io.pred.cols)[19]<- "GGT"

names(mys.io.pred.cols)[18]<- "Chloride"

names(mys.io.pred.cols)[17]<- "ALT"

names(mys.io.pred.cols)[16]<- "Calcium"

names(mys.io.pred.cols)[15]<- "Bilirubin"

names(mys.io.pred.cols)[14]<- "Basophil"

names(mys.io.pred.cols)[13]<- "Albumin"

names(mys.io.pred.cols)[12]<- "Creatinine"

## Multi-colinearity test.

mlc.io<- multi.collinear(mys.io.pred.cols[,9:30], p = 0.05)

mlc.io

## Sodium.

## Hence we remove sodium from our data.

mys.io.cols<- mys.io.pred.cols[,c("SUBJID", "tumor_mut",

"Gender", "Liver_met", "ECOG_group",

"pdl1_group", "histology",

"race_group", "AGE","BLWGHT", "Baseline_sld",

"Creatinine",

"Albumin", "Basophil", "Bilirubin", "Calcium",

"ALT", "Chloride", "GGT", "Eosinophil",

"Glucose", "LDH", "NLR",

"Monocyte", "Magnesium", "Platelet", "Potassium",

"Hemoglobin", "AST", "IMAE_Response")]

## 605 obs from 30 vars.

## Check for variables with near zero variance.

nzv.io<- nearZeroVar(mys.io.cols[,9:29])

## None.

table(mys.io.cols$IMAE_Response) ## NO: 395, YES: 210

prop.table(table(mys.io.cols$IMAE_Response))

## NO: 65%, YES: 35%.

## Data balancing.

set.seed(11)

d.ov<- ovun.sample(IMAE_Response~.,data = mys.io.cols,

method = "over",

N=790)$data

table(d.ov$IMAE_Response) ## No: 395, Yes: 395.

d.ov$IMAE_Response<- as.factor(d.ov$IMAE_Response)

##---------------------------------------------------

##----------------Model building----------------------------

set.seed(22)

mys.rf.io.ov<- randomForest(IMAE_Response~AGE+BLWGHT+Baseline_sld+Creatinine+

Potassium+Magnesium+

Glucose+Chloride+Calcium+

Platelet+Monocyte+Bilirubin+

Hemoglobin+GGT+Basophil+

Albumin+Eosinophil+

AST+ALT+LDH+NLR+race_group+

histology+pdl1_group+tumor_mut+

ECOG_group+Gender+Liver_met,

data = d.ov,

importance = TRUE,

proximity = TRUE)

print(mys.rf.io.ov)

## oob error: 12.28%.

## Feature Selection.

png(

'top10_features.tiff',

height = 16,

width = 20,

units = 'cm',

res = 600

)

varImpPlot(mys.rf.io.ov, sort = TRUE, n.var = 10,

type = NULL, class = NULL,

scale = TRUE,

main = "Top Ten Features")

dev.off()

## Top 10 imp features:

## cal, ldh, ggt, nlr, mono, plat, alt, gluc, sld, eos.

## Model building using top 10 features.

set.seed(555)

predictive_model<- randomForest(IMAE_Response~Calcium+

LDH+GGT+NLR+

Monocyte+Platelet+ALT+

Glucose+Baseline_sld+

Eosinophil, data = d.ov,

importance = TRUE,

proximity = TRUE)

predictive_model

## OOB ERROR: 14.3%.

plot(predictive_model)

png(

'oob_ov_topten.tiff',

height = 16,

width = 20,

units = 'cm',

res = 600

)

plot(predictive_model)

dev.off()

oob.err.data.topten <- data.frame(

Trees = rep(1:nrow(predictive_model$err.rate), 3),

Type = rep(c("OOB","YES","NO"), each = nrow(predictive_model$err.rate)),

Error = c(predictive_model$err.rate[,"OOB"], predictive_model$err.rate[,"YES"], predictive_model$err.rate[,"NO"]))

png(

'topten_error.tiff',

height = 16,

width = 20,

units = 'cm',

res = 600

)

ggplot(data = oob.err.data.topten, aes(x = Trees, y= Error)) + geom_line(aes(color = Type))

dev.off()
